# Design of a Wearable Haptic Device for Hand Palm Cutaneous Feedback

**DOI:** 10.3389/frobt.2021.706627

**Published:** 2021-09-07

**Authors:** Mihai Dragusanu, Alberto Villani, Domenico Prattichizzo, Monica Malvezzi

**Affiliations:** ^1^ Department of Information Engineering and Mathematics, University of Siena, Siena, Italy; ^2^ Department of Advanced Robotics, Istituto Italiano di Tecnologia, Genova, Italy

**Keywords:** cutaneous stimuli, force feedback, device design, hand palm, wearable haptic device

## Abstract

This study describes the main design and prototyping steps of a novel haptic device for cutaneous stimulus of a hand palm. This part of the hand is fundamental in several grasping and manipulation tasks, but is still less exploited in haptics applications than other parts of the hand, as for instance the fingertips. The proposed device has a parallel tendon-based mechanical structure and is actuated by three motors positioned on the hand’s back. The device is able to apply both normal and tangential forces and to render the contact with surfaces with different slopes. The end-effector can be easily changed to simulate the contact with different surface curvatures. The design is inspired by a smaller device previously developed for the fingertips; however, in the device presented in this study, there are significant differences due to the wider size, the different form-factor, and the structure of hand palm. The hand palm represents the support for the fingers and is connected to the arm through the wrist. The device has to be developed taking into account fingers’ and wrist’s motions, and this requirement constrains the number of actuators and the features of the transmission system. The larger size of the palm and the higher forces challenge the device from a structural point of view. Since tendons can apply only tensile forces, a spring-based support has been developed to keep the end-effector separated from the palm when the device is not actuated or when the force to be rendered is null. The study presents the main design guidelines and the main features of the proposed device. A prototype has been realized for the preliminary tests, and an application scenario with a VR environment is introduced.

## 1 Introduction

Nowadays technology is increasingly present in our everyday lives, and among the emerging technologies those oriented to the reproduction of tactile, kinesthetic, and skin sensations are getting interest in several application fields. These technologies allow enriching the sensory experience of humans, for instance in virtual environments, in augmented or mixed-reality applications, and/or during teleoperation tasks. Recent studies available in literature confirm that the use of tactile technologies not only increases the involvement of users in teleoperation tasks but also increases their accuracy and performance (Abbott et al., 2007; Battaglia et al., 2011). Interesting applications of haptic technologies are also present in telemedicine and in tele-rehabilitation, these applications have become particularly significant since the beginning of the past year, when the pandemic situation required solutions for guaranteeing social distance and human/human physical contacts.

The hand is one of the primary interfaces connecting humans and the surrounding environment, and it is also one of the main targets of haptics technology development. Most of the haptic devices for tactile stimuli are located on the fingers or on the wrist, while fewer are developed specifically for the palm (Pacchierotti et al., 2017); although Klatzky and Lederman’s studies demonstrated that the hand-closing task depends on haptic information in the palm (Lederman and Klatzky, 1987). Haptic devices developed for the hand palm can be broadly divided in two types: the grounded ones, having a base external to the user’s body and connected to a fixed base; and the wearable ones. Concerning grounded devices, for instance, in Iwata et al. (2001), the authors presented a device that can change the shape of the surface in contact with the hand using vertical motions of some pistons, while in Borst and Cavanaugh (2004) an array of actuators returns vibrotactile stimuli on users’ palm. In Martinez et al. (2018), a grounded planar device returns haptic stimuli and several sensations on users’ palms, and the ultrasound technology allows us to provide stimuli on hand that is detached from the surface of the device, so that the hand is free to move in a wider workspace.

The research in this topic is increasingly oriented towards wearable devices to completely free the users and their workspace. To date, wearability is a fundamental requirement for haptic devices, and in this context the trade-off between the extension of the area of the palm that can be stimulated and the number of actuators used is almost inevitable (Pacchierotti et al., 2017).

In this study we present a haptic wearable device for rendering forces in the palm that attempts to find a trade-off between the aforementioned requirements. The state-of-the-art devices are mostly based on localized and fixed contact points on the palm. Among these, there are two main typologies of devices: devices based on vibrotactile actuations, and devices using mechanical actuation. The last ones usually employ tightening bands reproducing only a normal pressure on the palm, as presented in Minamizawa et al. (2008a). However, this kind of technology allows the user to feel a limited and predetermined type of sensations. Similarly Minamizawa et al. (2008b) presented a band with a mobile contact surface on the palm, while Achibet et al. (2015) showed a passive device composed by an elastic band to return distance hand/body by haptic feedback.

In Son and Park (2018a) and Son and Park (2018b) two haptics gloves with several mechanical active points are designed and developed: the first uses small rigid links, while the second is tendon actuated. The limit of these devices is the high number of actuators needed to reproduce haptic stimuli in an extended area as the human palm, for this reason the authors of Gollner et al. (2012); Martínez et al. (2014); Borja et al. (2018) chose vibrotactile matrices in contact with the palm to reduce the clutter of mechanical actuators, reducing, however, the similarity between the desired stimulus and the transmitted one. A combination of mechanical stimuli and vibrotactile stimuli was shown by Kovacs et al. (2020) where a mobile mass comes in touch with a palm in case of collision between a hand avatar and the surrounding environment in virtual reality; while pneumatic solutions were presented by Kajimoto (2012) and Zubrycki and Granosik (2017). The authors in de Tinguy et al. (2020) presented a wearable haptic interface for natural manipulation of tangible objects in virtual reality that render contact force using a sensorized mobile mass grounded on wrist.

Haptic devices with mobile and an orientable contact area such as those presented by Trinitatova et al. (2019) and Trinitatova and Tsetserukou (2019) are closer to what we propose in this study for what concerns the design; however, in this study we want to investigate the possibility of reducing the footprint in the palm and the overall mechanical load on the hand through the use of cables for the transmission of forces.

In order to summarize and clarify the solutions available in the literature, Table 1 shows the details of the implementation and electronics of the devices mentioned above and of the device presented in this study.

**TABLE 1:** Comparison between the proposed haptic device (indicated as HaptiPalm, last row) and other solutions available in the literature.

Name	Type of actuation	Contact area	Form factor of electronics
Ghostglove (Minamizawa et al., 2008a; Minamizawa et al., 2008b)	Squeezing band	Fixed and extended	On board
Achibet et al. (Achibet et al., 2015)	Passive elastic band	Fixed and point-like	–
Son et al. (Son and Park, 2018a)	Four pressure points	Fixed and point-like	On arm
Son et al. (Son and Park, 2018b)	10 pressure points tendon drive	Fixed and point-like	Delocalized
Gollner et al. (Gollner et al., 2012)	35 vibrating motors	Fixed and point-like	On arm
Martınez et al. (Martínez et al., 2014)	Six vibrating motors	Fixed and point-like	Delocalized
Borja et al. (Borja et al., 2018)	Five vibrating motors	Fixed and point-like	On arm
Haptic pivot (Kovacs et al., 2020)	Mobile vibrating mass	Fixed and extended	on arm
Zubrycki et al. (Zubrycki and Granosik, 2017)	10 pneumatic pad	Fixed and extended	Delocalized
Weatavix (de Tinguy et al., 2020)	Mobile mass	Fixed and extended	On arm
Deltatouch (Trinitatova et al., 2019; Trinitatova and Tsetserukou, 2019)	3D Mobile platform	Mobile and point-like	Delocalized
HapticPalm	3D Mobile platform tendon drive	Mobile and interchangeable	On board

The device presented in this study is able to stimulate a large area of the palm with a limited number of actuators (three), therefore assuring a good wearability. In order to achieve these objectives a parallel mechanism has been designed in which the mobile platform (end-effector) has interchangeable contact interfaces. The various contact interfaces, having different shapes, can be easily connected and disconnected to/from the device to reproduce a tactile sensation more similar to the desired stimulus in different tasks.

Inspired from a family of wearable devices that we developed for fingertip stimulation (Chinello et al., 2018; Chinello et al., 2020), the mobile platform has a “Y” shape, and it is actuated by three servomotors positioned on the back of the hand by means of tendons, allowing the transmission motion. Notwithstanding the kinematic structure of the device presents some similarities with the devices previously presented for the fingertips; the solicitation of hand palm presents several different challenges, due to the different size, the kinematic structure, the form factor, and the force level. In order to provide to the user the possibility to easily wear the device and avoid the continuous contact with the palm, we added an elastic element at the base of the platform. The tendons and the springs allow us to reproduce the cutaneous stimulus both in static and dynamic conditions. The use of three actuators, furthermore, allows reproducing both normal and tangential components of the contact force (necessary for simulating shear forces) and the contact with virtual/remote surfaces with different orientations (that can be used for instance for reproducing the contact with variable curvature surfaces).

**FIGURE 1:**
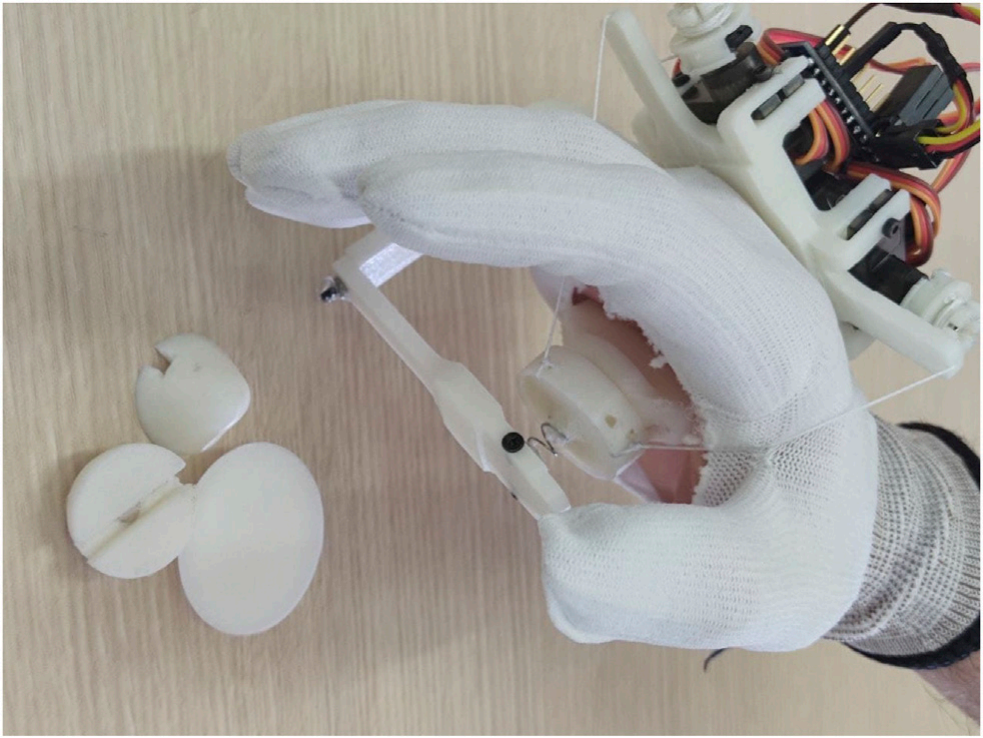
Prototype of the wearable haptic device for hand palm stimulation worn by a user.

The wearable haptic device for hand palm stimulation is shown in Figure 1. In particular, in this study, we will: 1) describes the haptic device for hand palm stimulation based on tendons, designed taking into account the physical/anatomical features of the palm; 2) presents the design of interchangeable modules for simulations of different types of contact; 3) details the mechanical, mechatronics, and manufacturing aspects of the device, including the finite element method (FEM) analysis, hardware, and control description; and 4) presents a working prototype of the device with some preliminary applications.

## 2 Biomechanics and Perceptive Receptor of the Palm

The design of the wearable haptic device started from the analysis of the hand palm: its biomechanical structure and the features of the stimuli to be rendered define the requirements and the constraints. From an anatomical point of view, the palm of the hand is the ventral (or anterior) region of the hand, the one to which the fingers converge when punching. The back of the hand, on the other side, is the posterior region of the hand, located from the opposite side of the palm. The bones of the palm can be divided into carp bones and metacarpal bones. The metacarpus is the set of five long bones that connect the carpus to the phalanges, and they are numbered from 1 to 5 starting from the thumb to the little finger. The carpus consists of eight short bones spread over two rows, the proximal and the distal row, and connects the radio with the metacarpus.

**FIGURE 2:**
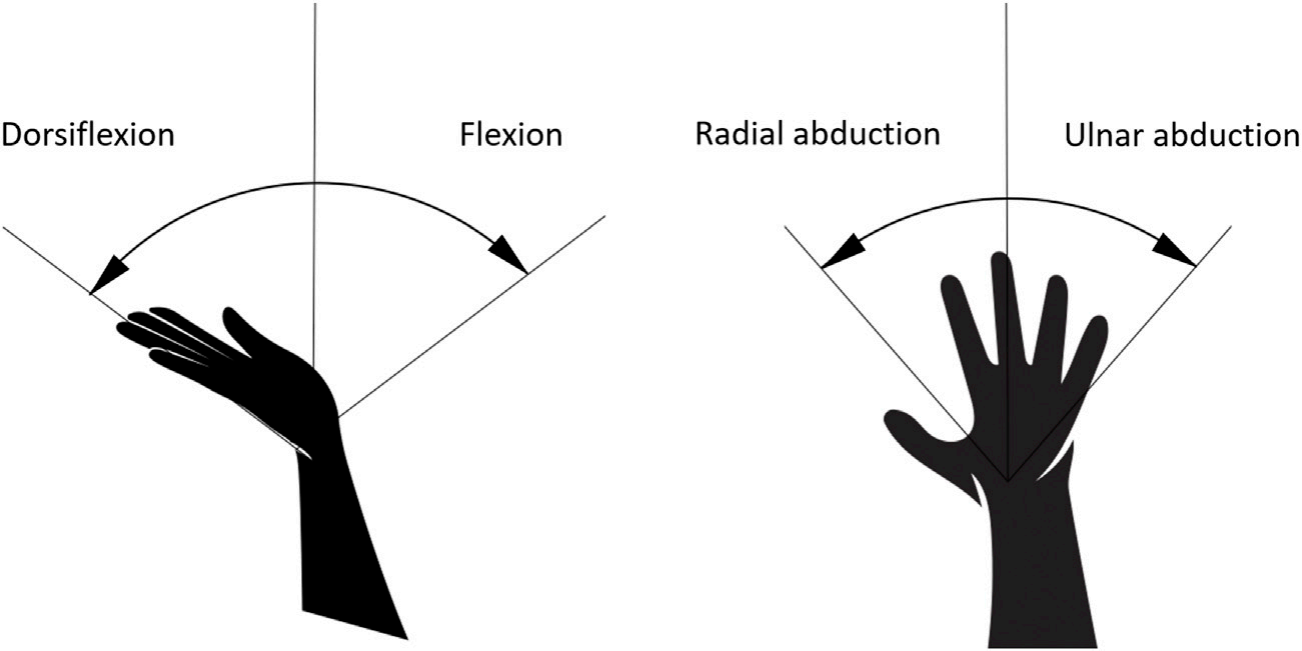
Primary hand movements due to the biomechanical structure of the carpus: palmarflexion/dorsiflexion **(left)** and radial abduction/ulnar adduction **(right)**.

The proximal row is composed by scaphoid, lunate, triquetrum, and pisiform bones, while the distal row is defined by trapezium, trapezoid capitate, and hamate bones. The biomechanical structure of palm bones, muscles, and ligaments allows the radial abduction, the ulnar adduction, the palmar flexion/dorsiflexion, and combined movement, all depicted in Figure 2. According to Swartz (2002), the palmar flexion is the flexion of the wrist towards the palm and ventral side of forearm, while the dorsiflexion is the hyperextension of the wrist joint, towards the dorsal side of forearm; radial abduction is a motion that pulls a structure or part away from the midline of wrist and ulnar adduction is a motion that pulls the hand structure toward the midline of wrist. Other two principal motions are provided by the biomechanical structure of the palm: the opposition and apposition of thumb. In apposition, the side part of thumb is in touch with other fingers, while the pulp side of thumb distal is in contact with fingertips of other digits during opposition (Van Nierop et al., 2008).

The contact surface of the developed haptic device has been dimensioned and placed according with biomechanics of palm, avoiding cluttering of palm and the reduction of carpus mobility. The mobile platform should not constrain and limit any motion described above; it should be placed under metacarpal bone *in situ* and extended from upper bound of first row of carpal bone to transverse metacarpal ligament.

The articulation of the bones of the carpus constitutes, on the handheld side, a cavity called the carpal tunnel. The carpal tunnel develops a flywheel on the wrist and is crossed by the superficial and deep flexor muscles of the fingers and the long flexor of the thumb. The median nerve also runs through the carpal tunnel, and its palmar branches, called digital cutaneous branches or cutaneous digital nerves, are distributed to the palmar skin up to the first three fingers (thumb, index finger, and middle). The palm, like the fingertips, is one of the densest regions of the human body of mechanoreceptors. Then channel of haptic stimulus transmission is the same for fingertips and palm. However, biomechanics of palm is more complex than finger one, as the geometry, anatomic constrains, and mechanical compliance (Kubo et al., 2018) are different, and the increased extension of the device is the cause of greater structural fragility, while the forces to render on palm are higher and, consequently, the structural stress on the device is greater too.

## 3 Haptic Stimulus of the Hand Palm: Design Principles and Modeling

The proposed device is able to both push the end-effector towards the palm and differentiate the applied force direction, contact location, and the orientation, ensuring robustness, ergonomics, and low weight. To achieve these requirements, the end-effector is passively supported by a spring connected to a C-shape structure fixed on one side on the hand’s back. The end-effector is then moved by three tendons actuated by three motors positioned on the hand’s back. Both the part fixed on the hand’s back and the active end-effector has a Y-shape, whose vertices are connected by three tendons actuated by three motors. This structure allows to apply a wide set of movements to the end-effector and to interact with most of the palm surface. In the following we will introduce a simplified mathematical model that relates with the force applied to the hand palm to the forces applied by the motors to the tendons.

The problem of cable driven parallel mechanisms is an interesting topic studied by several researchers in robotics context. Using cables and tendons to transmit movements allows designers and engineers to obtain compact and small-size devices, reducing the weight and inertial effects of mechanical components. The solution of the direct geometric-static problem of three cable-driven parallel robots by interval analysis is presented in Berti et al. (2013), while the case of direct geometric-static analysis of an underconstrained 4-cables parallel robot is presented in Carricato and Abbasnejad (2013). In Miermeister et al. (2013) the differential kinematics is studied for calibration, system investigation, and force-based forward kinematics. Moreover, the dynamic modeling of cable-driven parallel robots for a fully constrained planner case is investigated in Khosravi and Taghirad (2013) and for an underconstrained spatial case is presented in Yao et al. (2013).

Solving the forward kinematics problem means finding the relationship between lengths of the cables and posture of the moving platform; this information is important for the device control, but it is not easy to be solved in parallel mechanisms. On the other hand, the inverse kinematics problem evaluates the lengths of the cables corresponding to a given platform posture. Static analysis defines the relationship between cable tensions and wrench exchanged with the palm.

**FIGURE 3:**
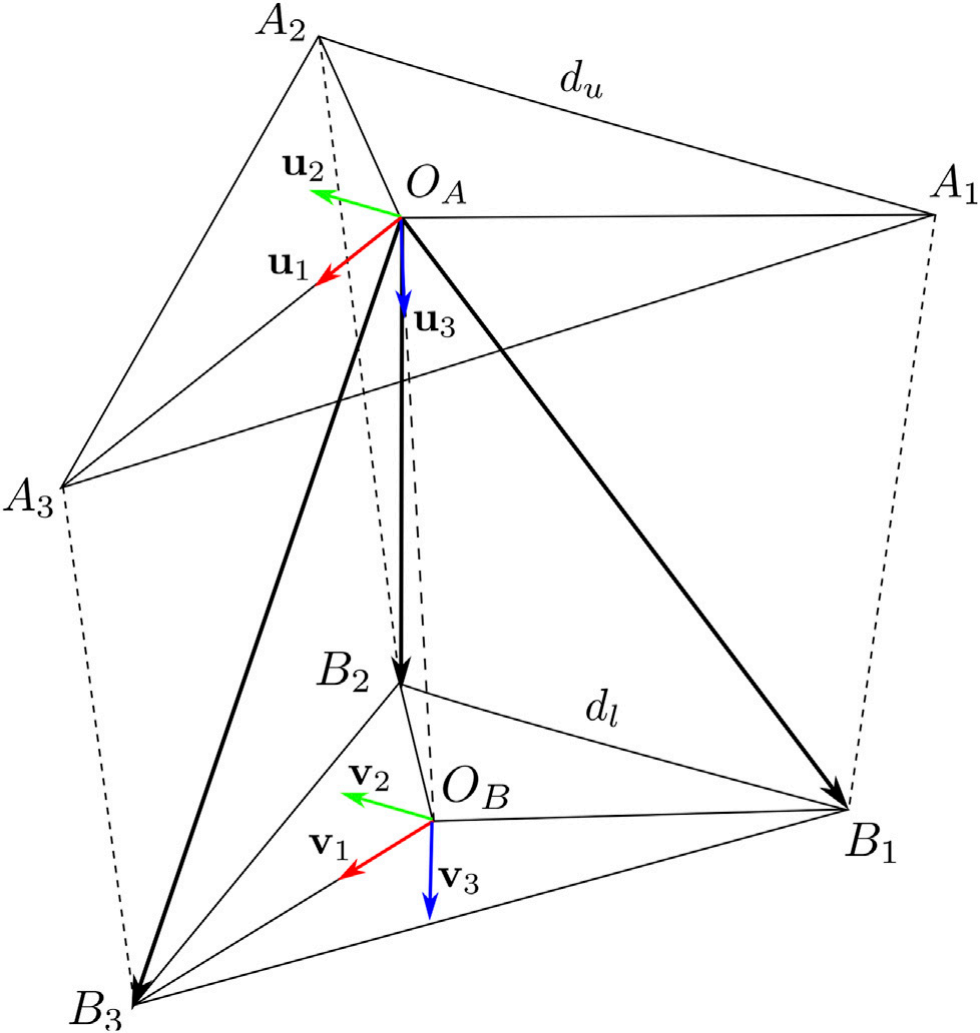
Main points and coordinate frames for the analysis of the wearable haptic device.

To describe the device as a cable-driven parallel robot, two coordinate frames are defined. The first one $A=\left\{O_{A},u_{1},u_{2},u_{3}\right\}$ is fixed on the device body on the hand back, while the second one $B=\left\{O_{B},v_{1},v_{2},v_{3}\right\}$ is fixed on the mobile platform in contact with hand palm, as shown in Figure 3. In the following, to simplify the notation, if quantities are expressed in A frame, the superscript A is omitted.

On the device body we define three connection points *A*_1_, *A*_2_, *A*_3_, representing the points where the tendons pass through in the device-fixed part; assuming they define an equilateral triangle, their coordinates are:

$$\begin{array}{c} a_{1}={\left[\frac{\sqrt{3}d_{u}}{-6},\frac{d_{u}}{-2},0\right]}^{\text{T}}a_{2}={\left[\frac{\sqrt{3}d_{u}}{-6},\frac{d_{u}}{2},0\right]}^{\text{T}}a_{3}={\left[\frac{\sqrt{3}d_{u}}{3},0,0\right]}^{\text{T}} \end{array}$$ (1)

where *d*_*u*_ is the upper triangle side length. The wires are connected to the mobile platform in three points *B*_1_, *B*_2_, *B*_3_, defining an equilateral triangle with center *O*_*B*_, their coordinates in B frame are:

$$\begin{array}{c} b_{1}^{B}={\left[\frac{\sqrt{3}d_{l}}{-6},\frac{d_{l}}{-2},0\right]}^{\text{T}}b_{2}^{B}={\left[\frac{\sqrt{3}d_{l}}{-6},\frac{d_{l}}{2},0\right]}^{\text{T}}b_{3}^{B}={\left[\frac{\sqrt{3}d_{l}}{3},0,0\right]}^{\text{T}} \end{array}$$ (2)

where, *d*_*l*_ is the length side of lower triangular plate, while their coordinates in A reference frame are indicated as:

$$\begin{array}{c} b_{1}={\left[x_{1},y_{1},z_{1}\right]}^{\text{T}},b_{2}={\left[x_{2},y_{2},z_{2}\right]}^{\text{T}},b_{3}={\left[x_{3},y_{3},z_{3}\right]}^{\text{T}}, \end{array}$$

and vary according to the platform motion. The coordinates of *O*_*B*_ point, expressed in A frame, are defined by the vector **o**_*B*_ = [*x*,*y*,*z*]^T^.

Let us define a rotation matrix **R**(***η***) = **R**_*z*_(*ϕ*)**R**_*y*_(*θ*)**R**_*x*_(*ψ*) representing the orientation of B frame w.r.t. A frame. We indicate with $\eta ={\left[\begin{array}{c} \psi ,\theta ,\phi \end{array}\right]}^{\text{T}}$ a vector containing roll, pitch, and yaw angles, respectively.

### 3.1 Inverse Kinematics

The inverse kinematic problem consists in finding the values of cable lengths *l*_*i*_ for a given position **o**_*B*_ and orientation **R**. The geometric constraints of the parallel structure relate to the length of the cables to the norm of the geometric vectors connecting *B*_*i*_ to *A*_*i*_, that is,

$$\|b_{i}-a_{i}\|=l_{i}\;\;i=1,2,3$$ (3)

where *l*_*i*_ is the length of cable *i* that can be controlled by the motors. The coordinates of the mobile platform connection points can be evaluated as:

$$b_{i}=o_{B}+Rb_{i}^{B}\;\;i=1,2,3$$ (4)

By substituting (Eq. 4) in the cable constraint relationship (Eq. 3), we can easily evaluate *l*_*i*_ as a function of **o**_*B*_ and **R**.

### 3.2 Statics

In stationary conditions, the sum of the forces and torques (wrench) applied to the platform through the wires is balanced by the forces and torques (wrench) due to the physical contact with the finger pad as follows:

$$\begin{array}{l} \overset{3}{\underset{i=1}{\sum }}f_{i}+f_{o_{B}}+f_{s}=0 \end{array}$$ (5a)

$$\begin{array}{l} \overset{3}{\underset{i=1}{\sum }}\tau_{i}+\tau_{o_{B}}+\tau_{s}=0 \end{array}$$ (5b)

We indicate with $f_{i}\in R^{3}$ the forces applied by the cables, with $\tau_{i}\in R^{3}$ the corresponding momentum, with $f_{O_{B}}\in R^{3}$ the reaction force applied by the hand palm to the platform, with $f_{s}\in R^{3}$ the force due to spring deformation, with $\tau_{O_{B}}\in R^{3}$ the reaction torque, and with $\tau_{s}\in R^{3}$ the torque due to spring deformation, both expressed w.r.t. the *O*_*B*_ point. Expanding the equilibrium equations (Eq. 5) we get:

$$\begin{array}{l} \overset{3}{\underset{i=1}{\sum }}\rho_{i}u_{f_{i}}+f_{o_{B}}+f_{s}=0 \end{array}$$ (6a)

$$\begin{array}{l} \overset{3}{\underset{i=1}{\sum }}\rho_{i}b_{i}\times u_{f_{i}}+\tau_{o_{B}}+\tau_{s}=0 \end{array}$$ (6b)

where *ρ*_*i*_ is the tension cable *i*, and $u_{f_{i}}$ is the unit vector representing the cable direction, and can be evaluated as:

$$u_{f_{i}}=\frac{b_{i}-a_{i}}{l_{i}}$$ (7)

From (Eq. 6), and (Eq. 7), we can express (Eq. 5) in the matrix form:

$$\left[\begin{array}{ccc} u_{f_{1}} & u_{f_{2}} & u_{f_{3}}\\ b_{1}\times u_{f_{1}} & b_{2}\times u_{f_{2}} & b_{3}\times u_{f_{3}} \end{array}\right]\left[\begin{array}{c} \rho_{1}\\ \rho_{2}\\ \rho_{3} \end{array}\right]+\left[\begin{array}{c} f_{o_{B}}+f_{s}\\ \tau_{o_{B}}+\tau_{s} \end{array}\right]=0$$ (8)

which is well-known as geometric-statics equation of cable driven parallel robots (Berti et al., 2013).

## 4 Design and Analysis

The device presented in this study is the result of a trade-off between wearability, weight, and resistance to mechanical stress. The symmetrical geometry of the supports on the back of the hand and under the palm allows a homogeneous distribution of the forces applied by the motors and transmitted through tendons. One of the drawbacks of tendon-actuated devices is that the end-effector has to be always in contact with the hand and the tendons have to be stretched. To overcome this issue and allow the device to activate and deactivate the contact with the palm, the end-effector is connected to a C-shaped fixed element by means of an elastic element.

In the following, we describe with more details the device from the design point of view, the interchangeable end-effector’s modules that are in contact with the palm, and some results of a structural mechanical analysis.

### 4.1 HapticPalm

The device has a parallel structure and consists of two main parts: one on the back of the hand and the other below the palm, defined as the end-effector of the device.

**FIGURE 4:**
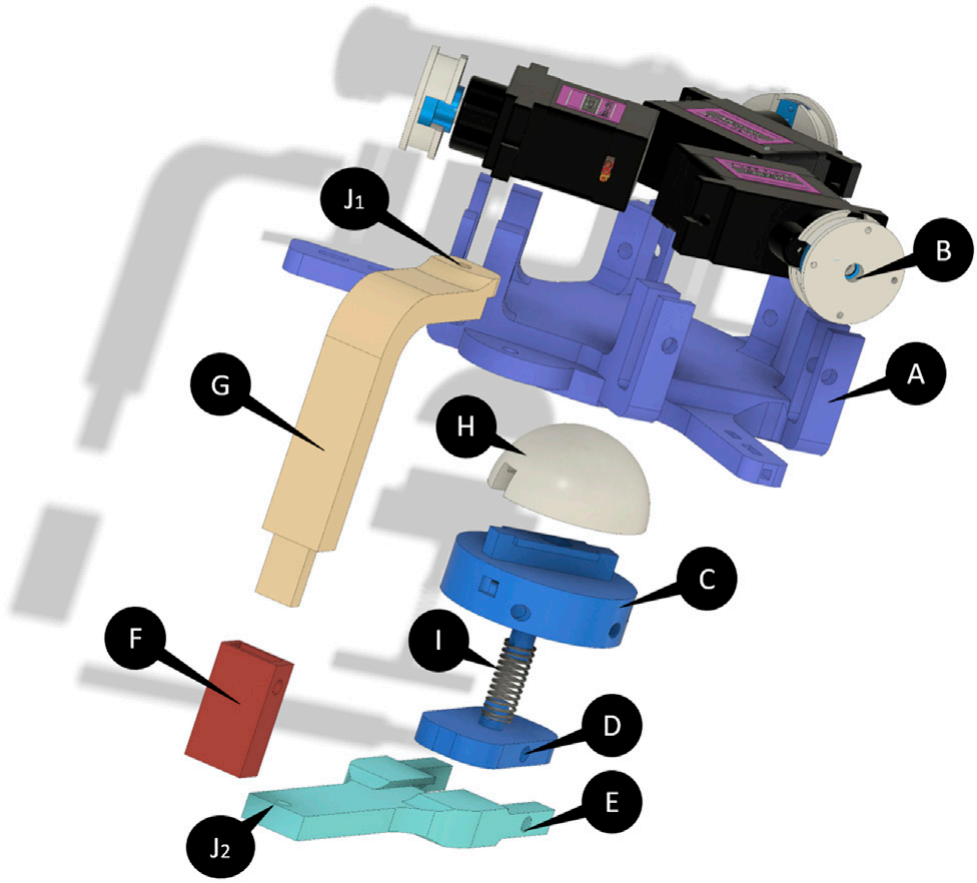
Exploded CAD view of the device. The end-effector of the device is composed of the components indicated with **(C,I,D)**. **(H)** indicates the end-effector’s module while the “Y-shaped” platform is indicated with **(A)** and the pulleys are indicated with **(B)**. **(E–G)** are the elements that realize the link between the two platforms. **(J**_**1**_ **)** and **(J**_**2**_ **)** are the two joints that allowed to rotate the end-effector.

The part on the hand’s back consists of a mechanical support for the force actuation/transmission system, the microcontroller, and the power supply (indicated with A in Figure 4). Three tendons are routed in three paths extruded from the support, arranged at 120°deg from each other in order to achieve an equilateral “Y-shape”. The tendons transmit the forces applied by the motors, through the pulleys, from the back of the hand to the device’s end-effector (B), and therefore to the palm. This type of actuation has an easy wearability and avoids the problems present in parallel mechanisms based on rigid links, that have higher weight, lower flexibility/adaptability, and requires suitable procedures to be adapted to different users and needs (Malvezzi et al., 2021).

The end-effector is composed of two platforms (D) and (C). The first is connected to the actuation system through the tendons, and the second is connected directly to the part on the back by means of a “C-shaped” rigid link (E). The two platforms are connected by an elastic element (a spring, I) that allows the tip-palm disconnection when no contacts and forces have to be applied. The connection points between tendons and the platform have a “Y-shaped” structure similar to the support on the back of the hand. A magnetic “T-shaped” interlocking system is designed on the first platform, allowing to easily interchange the different end-effectors’ modules (H) realized to reproduce different types of cutaneous stimuli.

The rigid link connecting the two main parts (G, F, E) has an adjustable telescopic height to adapt the device dimension to the needs of users with different anthropometric dimension of the hand, and can be rotated through two revolute joints (J1, J2), with the aim to ensure the ergonomics for several users and allow them to temporary move away the end-effector from the palm without remove the device.

### 4.2 Modules

**FIGURE 5:**
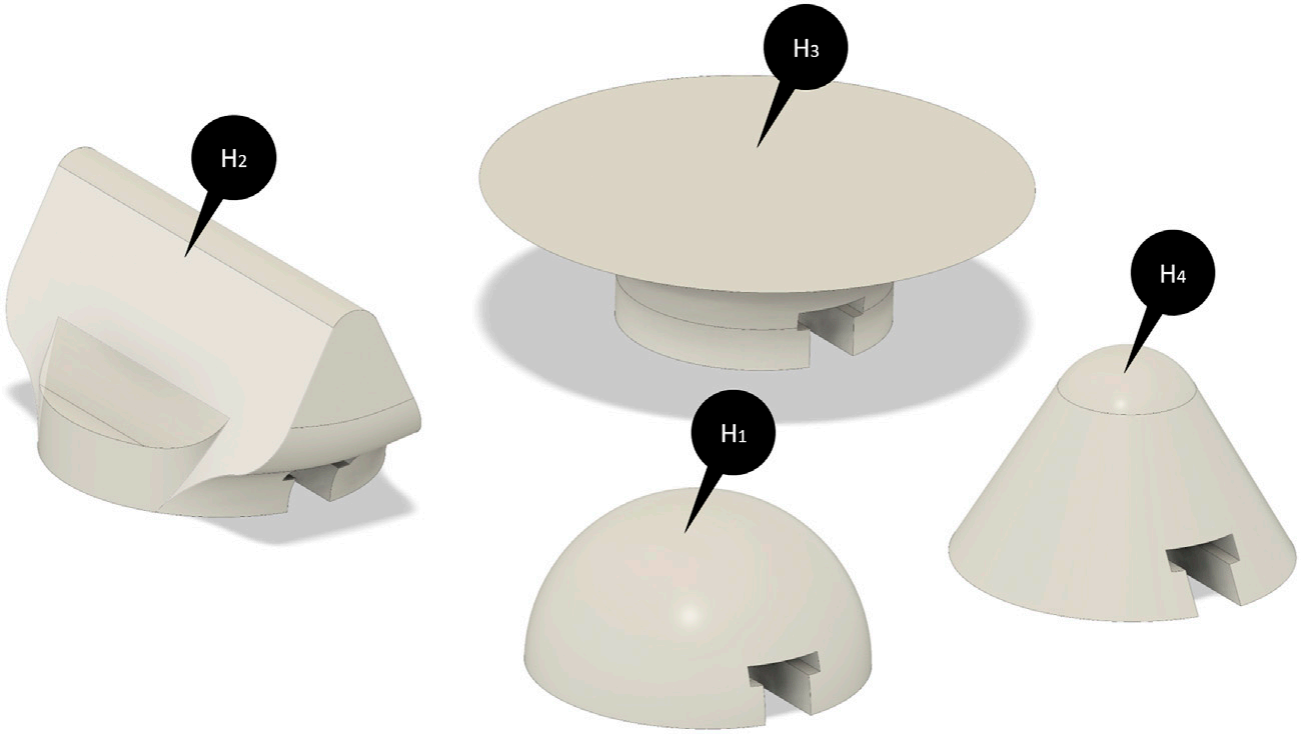
CAD models of the end-effector’ modules. (H_1_) and (H_4_) indicate the spherical modules with different radii of curvature while (H_2_) and (H_3_) are, respectively, the rectangular-shaped module and the plane-shaped module.

As mentioned before the end-effector modules of the device are manually interchangeable. They are fixed to the device by means of a “T-shaped” interlocking system. The modules can be connected/disconnected easily to the end-effector platform thanks to the “T-shaped” path and are fixed by a magnet inserted in each module. The magnets allow the modules to position themselves exactly in the center of the “Y-shaped” platform and to keep them fixed during the force rendering. In order to transmit the sensation of touching objects and surfaces that are interesting in haptics applications, four different end-effector modules have been created. The basic idea is to let the user feel common shapes such as spheres, corners, edges, and a flat surface. In this study, we have created two spherical modules with different radii of curvature with the aim to reproduce the curved surfaces (H1, H4 in Figure 5), a rectangular-shaped module and a plane-shaped module in order to reproduce the contact with edges and flat shapes, respectively (H2, H3).

### 4.3 FEM Analysis

Even if the basic structure of the device is similar to the one developed for the fingertip, for instance in Chinello et al. (2018), the larger dimensions and the magnitude of the applied forces are more challenging from the mechanical point of view. A structural stationary FEM analysis was carried out for evaluating the overall stress/deformation of the device in four different loading cases representing typical operative conditions. Three of them analyze the behavior of the device when a set of forces defined according to the model described in Section 3.2 are applied, while the fourth investigate the response of the device when the skin elasticity is saturated, that is, when the end-effector is fixed. The analysis was performed with 3D-CAD/CAE software, Fusion360 (Autodesk Inc., United States). The materials of the components used in these cases are ABS for all the components of the device except for the spring, realized in steel.

**FIGURE 6:**
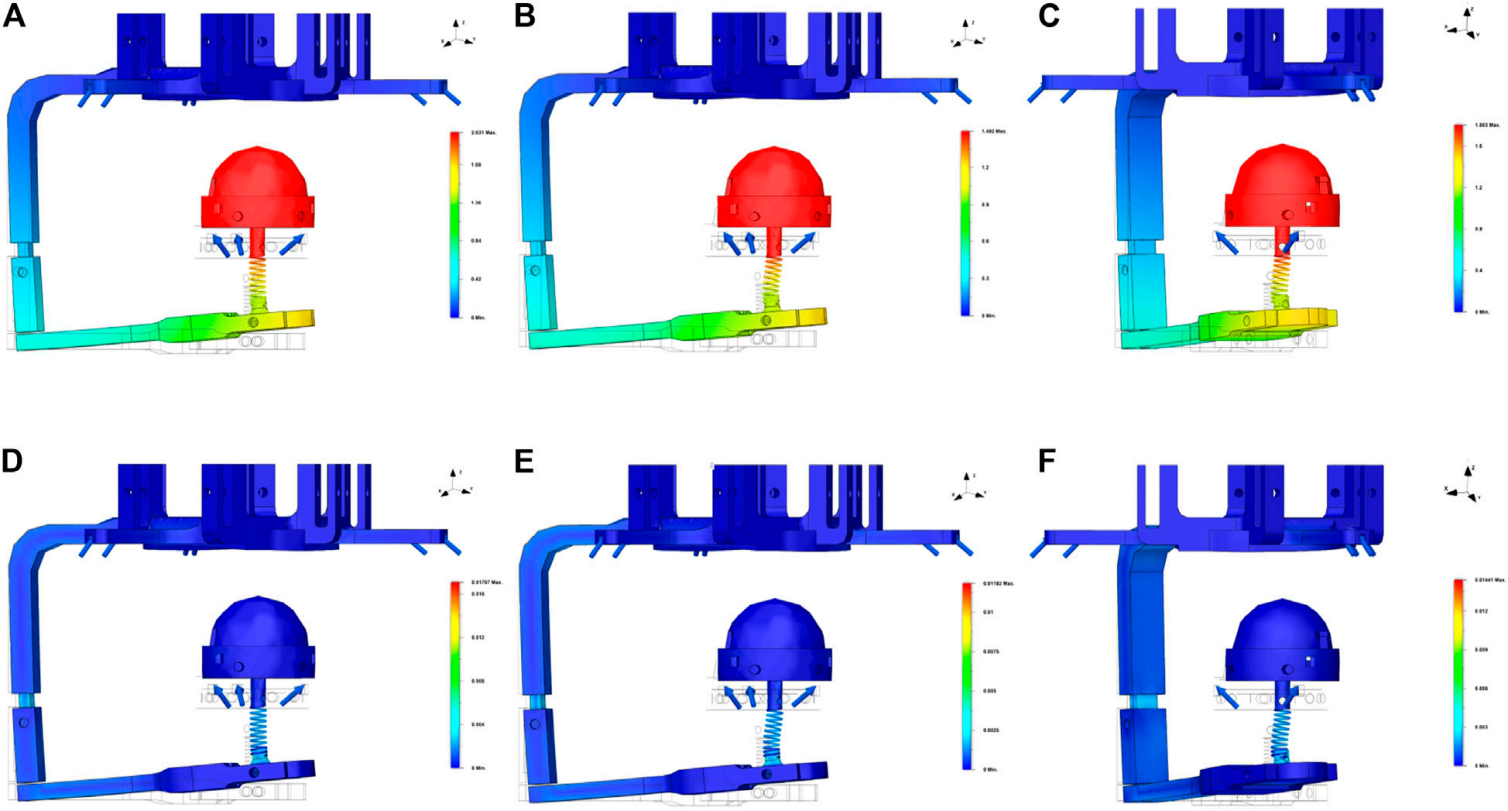
Results of the FEM structural analysis. The blue arrows indicate the forces applied in the simulations, the wireframe transparent CAD model represents the undeformed model, while a 1X deformation of the device is represented in the colored surfaces. **(A)** The displacements when a force with only a z-component is applied, **(B,C)** show, respectively, the results of the displacements when a force with a z-component and an x-component or a y-component is applied; subfigures **(D–F)** on the right side report the results of the deformation for the same loading cases.

The behavior of the device was analyzed when the forces indicated with blue arrows in Figure 6 are applied on the three vertices of both platforms. A fixed constraint was set on the bottom part of the back hand’s platform to simulate the contact with the hand. Six forces are applied to the fixed and mobile “Y” vertices, the directions of the forces are selected to simulate tendons’ actions. Their magnitudes are chosen according to the overall interaction force direction to be simulated.

Figure 6 shows the results of FEM analysis for the three-axis directions. All the subfigures on the top side report the results in terms of displacements while the subfigures on the bottom side show the results in terms of deformations. In Figures 6A, D, the results of the analysis corresponding to an overall force acting along the *z* direction are reported. In this case, the forces’ magnitudes are the same for all the simulated tendons and equal to 1 N. Concerning the corresponding displacement, Figure 6D shows that, as expected, it occurs mainly along the “z” axis.

Figures 6B, E show the results of the simulation of an overall force with components along the *z* and the *y* direction. For this simulation, we have symmetrically modified four forces, by reducing their module to 0.5 N.

In Figures 6C, F, we finally reported the results when an equivalent force with components along *x* and *z* directions is applied. As in the previous case, the forces have been modified symmetrically.

**FIGURE 7:**
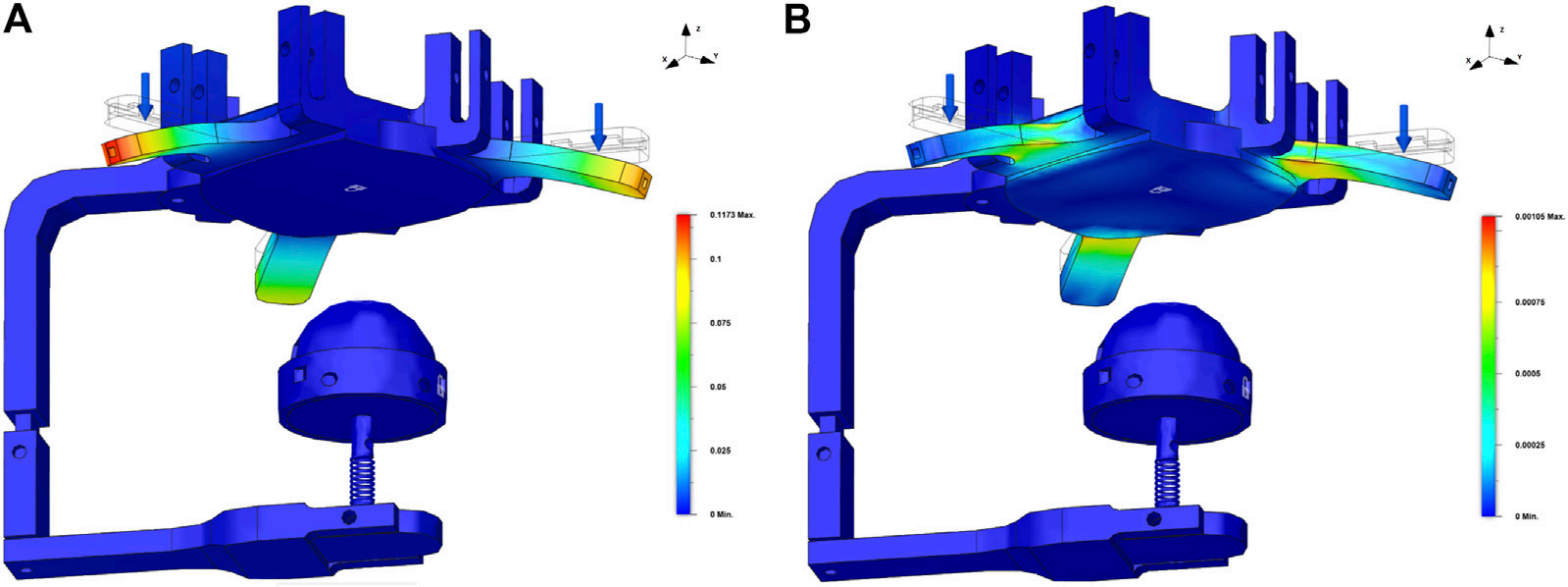
Results of the FEM analysis for the platform of the back of the hand under the action of an overall 15 N force (5 N for each side of the “Y-shape”). Blue arrows indicate the forces applied while the transparent wireframe, the CAD model, is the reference undeformed model, while the colored surface corresponds to a 1X deformation of the haptic palm device. Subfigure **(A)** shows the displacement of the platform and the subfigure **(B)** reports the deformation of the platform.

Finally, we verified the response of the device when the skin elasticity is saturated, to check the resistance of the platform on the back of the hand. The forces applied in this case are all with the same direction and intensity, that is, 5 N while the fixed constrains are two: one on the end-effector’s module and the other on the bottom part of the back hand’s platform (Figure 7).

In general, results from the FEM analysis shows that the mechanical structure of the device can resist to the forces that are applied in haptics applications. The results of FEM analysis also show that, as expected, the main critical points of the structure are represented by the rigid link and the base of the spring. Moreover, the choice of the spring is very another critical aspect to be considered to guarantee a suitable level of robustness and functionality of the device.

## 5 Prototype, Hardware and Components, Control, and Application

**FIGURE 8:**
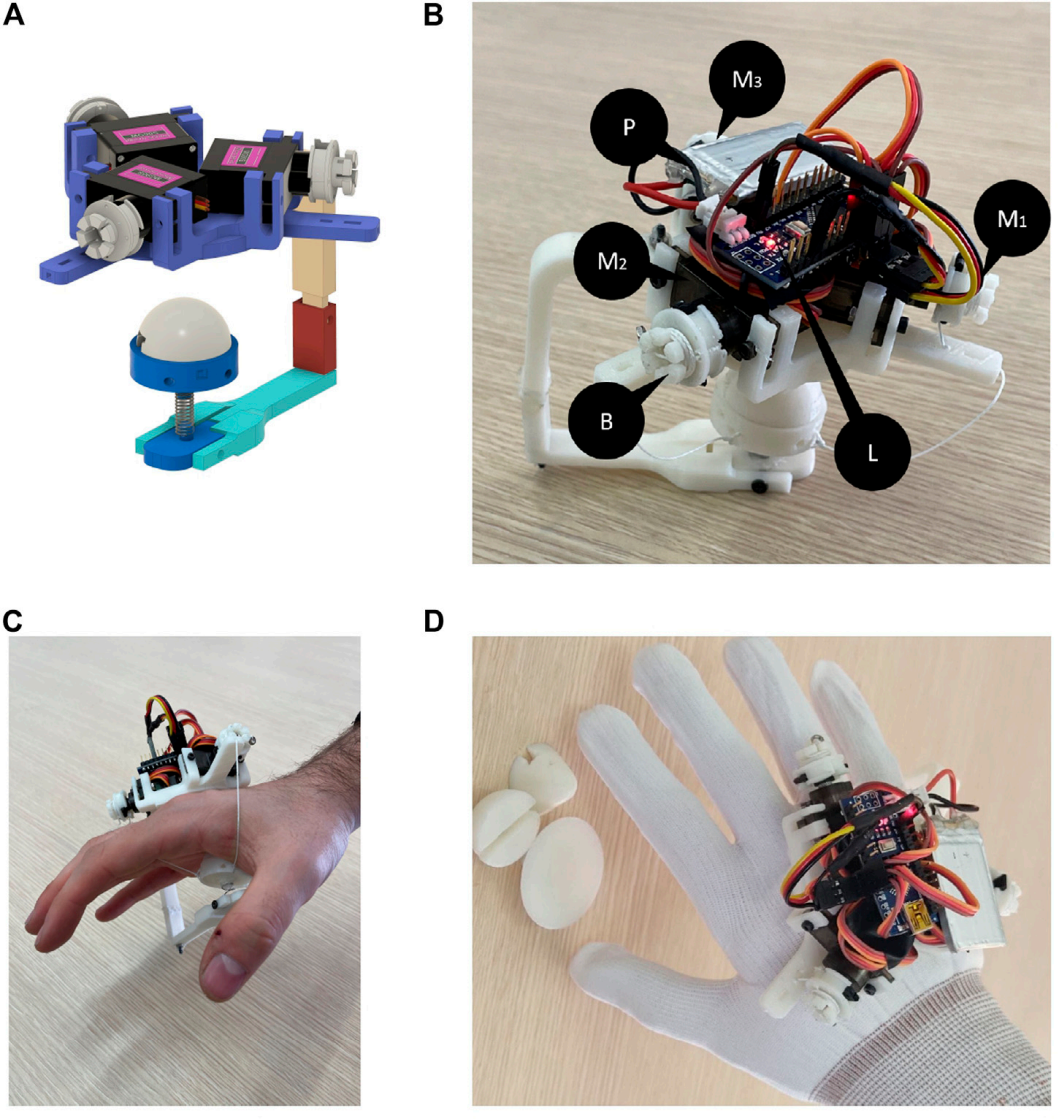
Prototype of the device presented in this study. Subfigure **(A)** shows the final CAD model of prototype, while **(B)** shows the device hardware components. Subfigure **(C)** report the “naked” device worn by a user. Subfigure **(D)** shows the final prototype worn by the user together with the different modules.

Figure 8 shows the first prototype of the developed device for hand palm haptics stimulation. All the structural components are manufactured using an FDM (fused deposition modeling) process with ABS material, except for the elastic element that is a standard off-shelf steel spring.

For the transmission of forces, three MG-90S microserves were used (M_*i*_ in Figure 8B), powered by a 5V battery (P). Each of them has a stall torque of 20 Ncm. For the control and data processing part, we used the Elegoo Nano V3+ microcontroller (ELEGOO Inc., CHN) (L), while the transmission with the virtual environment was carried out using serial communication. As previously introduced, the rendering of the force on the hand palm uses a tendon-based transmission. The tendons are anchored on one side to the pulleys of the motors fixed on the “Y-shaped” platform (A) of the back of the hand, on the other side to the “Y-arranged” connectors of the end-effector. The minimum size of each tendon is defined by the length of the thread needed, when it is under tension, to connect the platform of the end-effector to the “Y-shaped” platform on the back of the hand. In order to adjust this length, the pulleys have been designed with an external part that allows to wrap the excess amount of tendon. A first version of the pulleys is shown in the CAD model (B in Figure 4), while an updated version is shown in the prototype in Figure 8B.

Figure 8C shows the first prototype worn by a user. From the first users’ feedback, we realized that the “naked” device was not easy and intuitive to wear it due to the uncertainties on the orientation caused by the symmetrical shape of the device. Then with the aim to avoid this ambiguity in the orientation and to help/guide the user’s hand between the tendons when wearing the device, the device was embedded in a glove with a properly shaped hole in the center of the palm allowing the contact with the end-effector (Figure 8D).

As mentioned before, an elastic element was used in order to passively support the end-effector when it is not in contact with the palm. On one side the spring should be enough stiff to keep the end-effector in an upright position even when the tendons are not actuated. On the other side, the spring should be enough soft to avoid an overload to the motor, that should spend a part of their torque to apply the required haptic force and a part to deform the spring, as highlighted in Section 3.2. To meet this trade-off, we adopted a spring with a stiffness coefficient equal to 6.7 N/mm. Moreover, we decided to use a short spring, with a length of 7 mm, to keep the end-effector’s size as compact as possible and with this choice, in the prototype, we observed a symmetric behavior of the spring both along the axial and radial directions.

Table 2 summarizes the main characteristics of the developed prototype. The maximum and minimum theoretical forces that can be applied on palm are evaluated according to the model presented in Section 3.2.

**TABLE 2:** Haptic palm device prototype; main hardware/software details and technical specifications. The dimensions of device are measured without the glove, and the value of Δ*h* is equal to the capability to extend and shorten the rigid link F with respect to the G link.

	Technical specifics
Weight	89.92*g*
Dimensions *L* × *l* × (*h* ±Δ*h*)	104 × 81 × (97 ± 0.7)*mm*
Degrees of freedom	3
Microprocessor	*ATmega*328
Clock speed of microcontroller	16 *MHz*
Computer interface	*Serial COM*
Input voltage	5.0*V*
Battery life	≈5*h*
Maximum speed	$9.5\frac{rad}{s}$
Maximum resisting force	60.0*N*
Maximum theoretical force on palm	29.5*N*
Minimum theoretical force on palm	0.81*N*
Maximum recall force	30.5*N*
Maximum contact surface	≈7.06* cm* ^*2*^
Cost	≈ *$*50

### 5.1 An Example of VR Application

We have created a virtual reality scenario to demonstrate the functionality of the device in the case of different extended contacts. The scenario was developed in CoppeliaSim (Coppelia Robotics AG., CHE) and it is a replica of a common office station. In the simulated environment, it is possible to interact with a series of objects on a desk and with the desk itself, in detail, each object is designed to test a specific haptic device end-effector.

**FIGURE 9:**
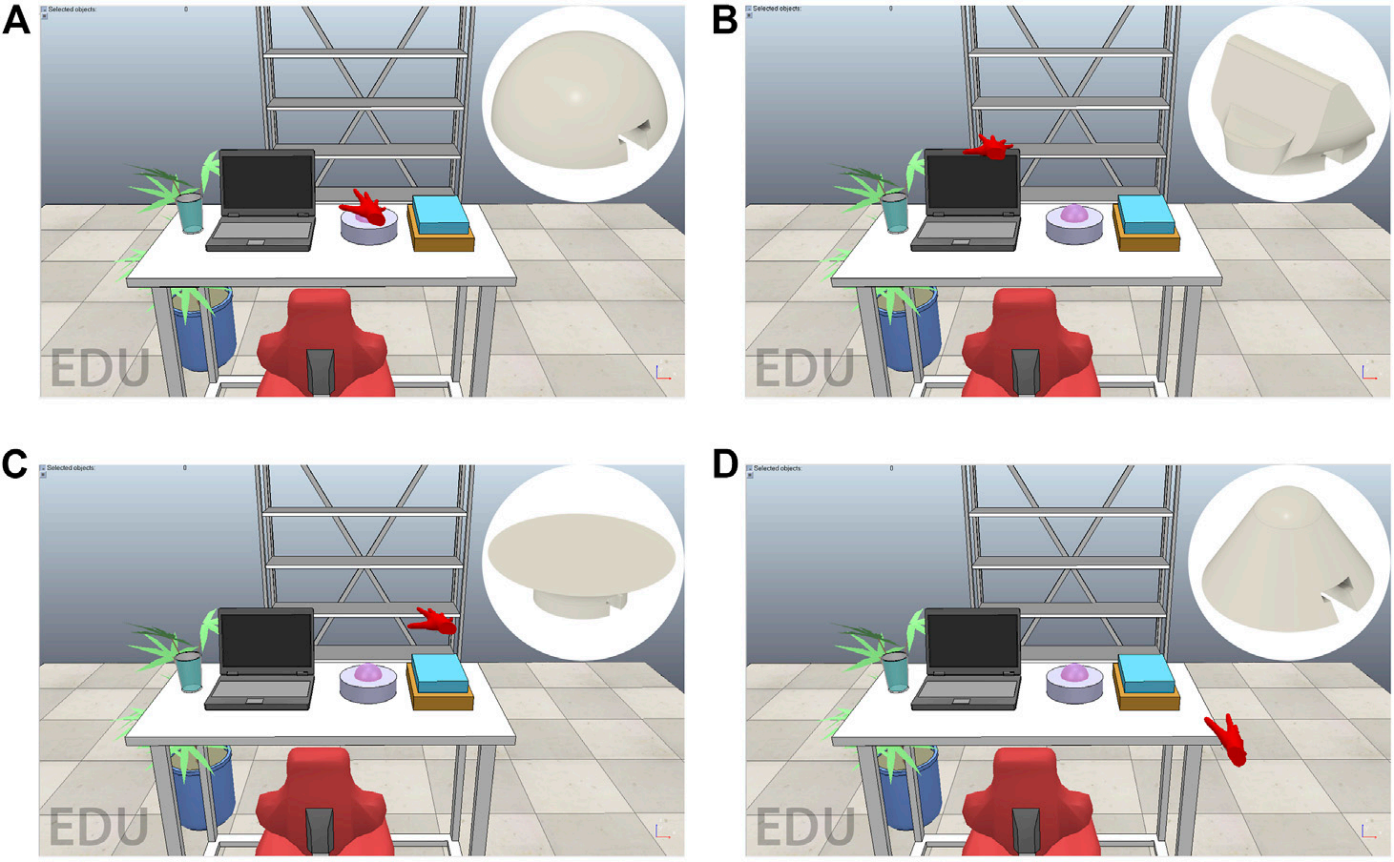
The testing virtual scenario is composed of a rack and a plant in vase in background and an office station in the foreground. The interaction is provided by the red hand avatar of and the objects in foreground are chosen to use all modules: paperweight for H1 module **(A)**; laptop frames for H2 module **(B)**; cover of book and the surface of desk for H3 module **(C)**; and desk, laptop, and book peaks for H4 module **(D)**.

The module H2 is suitable for the exploration of laptop frames (Figure 9B), and circular module H1 is intended for the interaction with the paperweight (Figure 9A), while the modules H3 and H4 are suitable for interacting with the desk plane and stacked books (Figures 9C,D). Interaction with these objects is possible through a hand avatar sensorized in the palm and controllable by LeapMotion controller (Ultraleap.inc., United States), a camera hand tracker system. As a further development of this study, an IMU-based tracking system will be integrated with the haptic device (Baldi et al., 2017). The forces, estimated by the triaxial virtual sensor, are the result of collisions with the dynamic model of the hand and objects, the collision forces are processed through a Bullet 2.78 physical engine. The contact forces are then transmitted to the device during navigation, and it is also possible to generate an on-demand signal from a recorded force profile from a previous exploration. The device and the virtual environment communicate *via* the serial port.^1^

## 6 Conclusion

This study introduces a wearable haptic device for a hand palm cutaneous stimulation, suitable to render the forces generated from palm/environment interactions in a virtual reality environment. As discussed in the initial part of the study, due to the biomechanical structure, constraints of the palm, and the need of wearability, the design of this device prototype is the result of different requirements, including limited dimensions, weight, capability to reproduce contact interactions, and force components in a large surface as the palm. Ideally, the device should be very light, should not over constrain the hand, should not encumber palm, and should apply forces suitable for accurately reproducing desired haptic simulations. The study proposes a tendon driven solution with a passive element that allows disconnecting the end-effector from the palm in absence of force to render.

The starting idea is a parallel tendon-driven mechanism actuated by three motors on the hand back. In the first part of the study, the mechanism has been studied from the theoretical point of view by means of a simplified mathematical model. Then the idea has been detailed and a CAD model of the device has been realized, and a FEM analysis has been conducted to simulate the behavior of device in case of skin elasticity saturation. A first prototype of the device has been realized and some preliminary functional tests have been conducted. Three servomotors actuate the tendons and transmit symmetrical force from a “Y-shaped” platform to an end-effector platform with a “Y-arrangement” of the connection points. Several modules have been designed and 3D-printed to extend set of the reproducible surface sensations. The design of modules allows the easy connection and disconnection by means of a “T-shape” socket and a magnetic clip. According to the first experiences of the users in a virtual environment and their opinions, the device was positioned and fixed on a shaped glove to guide the hand when wearing the device without covering the palm.

Future developments of this study will include the validation of forces provided by device, the change of serial communication on wire with a Bluetooth communication channel, an optimization of the design of some critical structural components, and the development of active interchangeable modules with sensors and/or multisensory actuators (e.g., thermal and vibrations) to increase capability of device in terms of precision and stimuli rendering, including cutaneous stimuli. In addition, we will conduct human studies on device usability and wearability for virtual reality applications.

## Data Availability

The original contributions presented in the study are included in the article/supplementary material; further inquiries can be directed to the corresponding author.

## Footnotes

1 A video demonstration is available at the following link: https://drive.google.com/file/d/15YgSfVR1ygYl6Do9mY12Ms9qyvuKcaSI/view?usp=sharing.
